# Collection de la cuisse révélant une fistule vésicale après radiothérapie pour cancer de la prostate

**DOI:** 10.11604/pamj.2018.30.197.16037

**Published:** 2018-07-05

**Authors:** Soufiane Ennaciri, Toufik Michel Bitard

**Affiliations:** 1 Service d’Urologie, Centre Hospitalier Universitaire Hassan II, Fès, Maroc; 2 Service de Chirurgie Viscérale et Urologique, Hôpital Simone Veil, Eaubonne, France

**Keywords:** Fistule vésicale, collection de la cuisse, cancer de la prostate, radiothérapie, cystographie, Vesical fistula, thigh collection, cancer of the prostate, radiotherapy, cystography

## Abstract

Urinary fistulas are a rare late complication after radiotherapy for prostate cancer. We here report the case of a 76-year old patient who had undergone radical prostatectomy 4 years before followed by external beam radiotherapy for prostate cancer. The patient presented with recurrent thigh swelling . Clinical examination showed renitent and painless mass at the level of the medial compartment of the right thigh. Ultrasound followed by CT scan showed big homogeneous fluid collection in the inner thigh exercising a slight compression on femoral vessels. Surgical drainage was performed removing 900 ml cloudy liquid whose bacteriological examination was negative. Post operative suites were marked by a sharp reduction of diuresis. However significant quantity of clear liquid continued to be drained. Given the patient's clinical picture, methylene blue test followed by retrograde cystography were performed confirming the diagnosis of fistula originating from the ureterovesical junction. The drainage of the collection was obtained through the insertion of a local probe for several weeks.

## Image en médecine

Les fistules urinaires sont une complication tardive rare après une radiothérapie pour le cancer de la prostate. Nous décrivons le cas d'un patient âgé de 76 ans, ayant eu quatre ans auparavant une prostatectomie radicale suivie d'une radiothérapie externe pour un cancer de la prostate. Le patient s'est présenté pour une tuméfaction de la cuisse récidivante. L'examen clinique a montré une masse rénitente et indolore au niveau de la partie médiale de la cuisse droite. Le bilan radiologique fait d'une échographie puis d'un scanner a objectivé une grosse collection liquidienne homogène de la face interne de la cuisse exerçant un léger effet de masse sur les vaisseaux fémoraux. Un drainage chirurgical a été réalisé ramenant 900ml de liquide louche dont l'examen bactériologique était négatif. Les suites post opératoires ont été marquées par une diminution brutale de la diurèse, par contre le drain a continué à ramener régulièrement des quantités importantes de liquide clair. Devant ce tableau, nous avons réalisé un test au bleu de méthylène puis une cystographie rétrograde qui ont confirmé qu'il s'agissait d'une fistule provenant de la jonction urétro-vésicale. L'assèchement de la collection a été obtenu grâce à un sondage à demeure pendant plusieurs semaines.

**Figure 1:**
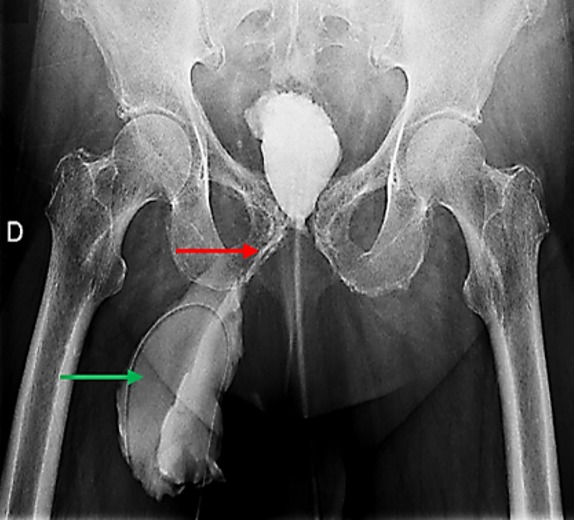
Cliché de cystographie rétrograde montrant le trajet fistuleux (flèche rouge) entre la vessie et la collection de la cuisse droite (flèche verte)

